# Alkylglycerols Modulate the Proliferation and Differentiation of Non-Specific Agonist and Specific Antigen-Stimulated Splenic Lymphocytes

**DOI:** 10.1371/journal.pone.0096207

**Published:** 2014-04-24

**Authors:** Linxi Qian, Mingshun Zhang, Shengmei Wu, Yan Zhong, Eric Van Tol, Wei Cai

**Affiliations:** 1 Shanghai Institute for Pediatric Research, Xinhua Hospital, Shanghai Jiao Tong University, School of Medicine, Shanghai, China; 2 Shanghai Key Laboratory of Pediatric Gastroenterology and Nutrition, Shanghai, China; 3 Department of Immunology, Nanjing Medical University, Nanjing, China; 4 Mead Johnson Pediatric Nutrition Institute, Shanghai, China; 5 Mead Johnson Pediatric Nutrition Institute, Nijmegen, the Netherlands; Hospital Nacional de Parapléjicos - SESCAM, Spain

## Abstract

Alkylglycerols (AKGs) are ether-linked glycerols derived from shark liver oil and found in small amounts in human milk. Previous studies showed that oral AKGs administration significantly increased the immune response in mice. The aim of the present study was to investigate the *in vitro* immunomodulatory effect of AKGs on stimulating splenic lymphocyte responses. C57BL/6 mice were immunized with hepatitis B surface antigen (HBsAg). Splenic B cells were purified and stimulated with anti-BCR and anti-CD38. Meanwhile, splenic CD4+ T cells were purified and stimulated with anti-CD3 and anti-CD28. For antigen specific stimulation, the purified CD4+ T cells were cocultured with HBsAg -pulsed dendritic cells. The stimulated lymphocytes were treated with different concentrations of AKGs. The cell proliferation was assessed by [^3^H]-thymidine incorporation assay. The maturation of B cells was assessed by examining the germline (GL) transcription of IgG (γ1) mRNA expression, and the surface expressions of CD80/CD86 markers were examined by flow cytometry analysis. Th1/Th2 polarity was assessed by T-BET (Th1)/GATA-3 (Th2) flow cytometry assay and by characteristic cytokines ELISA assay (TNF-α and IFN-γ for Th1; IL-4 and IL-10 for Th2). It was found that AKGs significantly increased the BCR/CD38 -stimulated B cell proliferation. The T cell proliferation in response to CD3/CD28 or specific antigen stimulation was also increased by AKGs. The transcriptional level of IgG (γ1) and the expressions of CD80/CD86 molecules were markedly increased by AKGs in BCR/CD38 -stimulated B cells. Meanwhile, the results showed that AKGs increased the expression of T-BET transcriptional factor and the production of Th1 cytokines (TNF-α and IFN-γ) upon CD3/CD28 stimulation; whereas, levels of Th2 cytokines (IL-4 and IL-10) were decreased by AKGs. Our study demonstrated that AKGs can modulate immune responses by boosting the proliferation and maturation of murine lymphocytes *in vitro*.

## Introduction

Shark liver oil (SLO) is purified from sharks that live in cold, deep oceans. It contains alkylglycerols (AKGs), squalene, pristane, lipid-soluble vitamins (A and D), Ω-3 fatty acids, triglycerides and other unsaturated fatty acids [1]. AKGs are ether-linked glycerols derived from SLO including such substances as batyl alcohol (AKG18/0), chimyl alcohol (AKG16/0) and selachyl alcohol (AKG18/1) [2]. AKGs are considered to be important immune stimulating factors, and are able to increase the number of leucocytes, lymphocytes and platelets. They can also be applied to treat leukemia, and even solid tumor [3]. Besides marine resources, AKGs are also synthesized in human body, and women need to synthesize more AKGs during lactation to meet the requirement of infant development. The previous study showed that human colostrum contains about 0.19% AKGs, while cow milk contains only 0.01% AKGs which accounts for 1/20 of those in human milk [4]. Clinical study showed that human milk provides advantages in terms of immunity, as well as significantly reduces the risk for a variety of chronic and acute diseases [5]. It was also demonstrated that human milk can enhance antibody response after vaccination [6]. Antibody production in response to immunization is lower in preterm and very low birth weight infants than in term normal birth weight infants due to their immature immune system [7]. Human milk is recommended for the immature infants to help the maturation of their immune system and boost their immune response to immunization [8]. AKGs, as one of the potent immune stimulators in nature, are believed to be important immunomodulatory factors in human breast milk. Previous study showed that oral AKGs administration increased vaccination-induced raise of specific immunoglobulin (Ig) in mice and pregnant sows [9], [10], [11]. Besides stimulating adaptive immune response, AKGs were demonstrated to have impact on innate immune response. Only a small dose of AKGs treatment greatly enhanced the Fc-mediated ingestion activity of macrophage in mice [12]. The enhancement of macrophage ingestion activity by AKGs treatment depends on the signaling crosstalk between macrophage and nonadherent cells (T and B cells), which indicates that the physiological roles of AKGs in immune system are contributed by the coordination of various immune cells.

Plenty of knowledge was known regarding to the immune action of AKGs *in vivo*, however, little was known about the effect of AKGs on the sensitized lymphocytes *in vitro* and the mechanisms by which AKGs boost immune response to immunization. To better understand the effects of AKGs on lymphocytes, we have focused on the role of AKGs in the proliferation and activation of splenic B cells upon BCR/CD38 stimulation. Since high yield of specific antibody in response to vaccination was known to be dependent on primed T cells, we also studied the impact of AKGs on the proliferation and activation of splenic T cells stimulated through CD3/CD28 or antigen-pulsed dendritic cells (DCs). Our results showed that AKGs can modulate immune responses by boosting the proliferation and maturation of murine lymphocytes *in vitro*.

## Materials and Methods

### Reagents

Batyl alcohol and chimyl alcohol were purchased from Bachem (Bubendorf, Switzerland). Hepatitis B surface antigen (HBsAg) used for animal immunization was from GlaxoSmithKline Biologicals. Antibodies used for B cell stimulation were anti–mouse BCR (115-006-075, Jackson ImmunoResearch) and anti-mouse CD38 (1635-01, Southern Biotechnology). Antibodies used for T cell stimulation were anti–mouse CD3 (16-0031-81, eBioscience) and anti-mouse CD28 (16-0281-86, eBioscience). B cell isolation kit was from Miltenyi Biotec (130-090-862). CD4+ T cell isolation kit (130-095-248) and CD4+CD62L+ T cell isolation kit (130-093-227) were from Miltenyi Biotec. FITC-anti-GATA-3 (130-100-689) and PE-anti-T-BET (130-098-653) were from Miltenyi Biotec. FITC-anti-CD80 (11-0801-86) and PE-anti-CD86 (12-0862-85) were from eBioscience. [^3^H]-thymidine was from GE Healthcare. Murine GM-CSF was from PEPROTECH (315-03). TNF-α, IFN-γ, IL-4 and IL-10 ELISA kits were from BD Biosciences.

### Immunization Procedures

The study was approved by the Animal Ethics Committee of Xinhua Hospital. C57BL/6 mice were purchased from SLAC Laboratories. The mice were housed in stainless steel cages with bedding (6 mice/cage). All the mice were exposed to a 12 -hour light and dark cycle, and fed a commercial laboratory chow (SLAC Laboratories). Ten adult (>8 wk) C57BL/6 mice were immunized subcutaneously with 5 µg HBsAg at day0, and reimmunized with 5 µg HBsAg at day7.

### Cell Isolation and Culture

Two weeks after reimmunization, spleens were collected for *in vitro* assays. Splenic naïve B cells were purified by depleting CD43+ B cells (activated B cells), T cells, NK cells, dendritic cells, macrophages, granulocytes and erythroid cells using negative selection beads against CD43, CD4 and Ter-119. Isolated B cells were cultured in RPMI 1640 medium supplemented with 10% heat-inactivated fetal bovine serum (FBS), 5×10^−5^ mol/L β-mercaptoethanol, 100 µg/ml streptomycin and 100 units/ml of penicillin in 5% CO_2_ incubator at 37°C.

For HBsAg specific stimulation, untouched CD4+ T cells were isolated from the splenocytes of immunized C57BL/6 mice using negative selection beads against CD8a, CD11b, CD11c, CD19, CD45R, CD49b, CD105, anti-MHC II and Ter-119. For non-specific stimulation, splenic naïve CD4+CD62L+ T cells were purified by depleting non-T helper cells, regulatory T cells and γ/δ T cells using negative selection beads against CD8a, CD45R, CD11b, CD25, CD49b, TCRγ/δ and Ter-119, and then using positive selection beads against CD62L. Isolated T cells were cultured in RPMI 1640 medium supplemented with 10% heat-inactivated FBS, 5×10^−5^ mol/L β-mercaptoethanol, 100 µg/ml streptomycin and 100 units/ml of penicillin in 5% CO_2_ incubator at 37°C.

### Lymphocyte Stimulation

For non-specific stimulation, splenic B cells were stimulated with the combination of anti-BCR and anti-CD38 (each 1 µg/ml). CD4+CD62L+ T cells were stimulated with the combination of anti-CD3 and anti-CD28 (each 1 g/ml). For HBsAg specific stimulation, bone marrow cells from tibias and femurs were plated in 12-well plates with RPMI 1640 medium with 10% heat-inactivated FBS and 10 ng/ml murine GM-CSF. At day 5, bone marrow cells were plated at 5×10^5^ cells/ml in 12-well plates and pulsed with 1 µg/ml HBsAg for 4 hours. To induce DCs maturation, cultures were treated with LPS (1 µg/ml) for 24 h. The DCs at day 7 were irradiated (5000 rad) and cocultured with CD4+ T cells at the ratio of 1∶10 [13]. Because cultures without costimulation generated only small numbers of viable cells, they were excluded from the analysis.

### Proliferation Assay

For [^3^H]-thymidine incorporation assay, lymphocytes in 96-well plates (2×10^5^ cells per well) were cultured with DMSO, batyl alcohol or chimyl alcohol (10 nM, 50 nM, 100 nM and 500 nM) in the presence of stimuli for 72 h. [^3^H]-thymidine (0.2 µCi/well) was added to cells for the last 4 h of culture, and the incorporation of [^3^H]-thymidine was determined by liquid scintillation spectrometry.

### Flow Cytometry

For intracellular molecular staining, T cells (2×10^5^) were fixed in 4% formaldehyde at 37°C for 10 min, and permeabilized in 90% methanol on ice for 30 min. After fixation and permeabilization, the cells were blocked in the incubation buffer (1% FBS in RPMI 1640 medium) at 37°C for 10 min, and then were cultured in incubation buffer with diluted (1∶100) FITC-anti-GATA-3 or PE-anti-T-BET at 37°C for 40 min. For surface molecular staining, B cells (2×10^5^) were directly blocked and cultured in incubation buffer with diluted (1∶100) PE-anti-CD86 and FITC-anti-CD80 at 37°C for 40 min. After incubation, the cells were rinsed twice and resuspended in PBS for analysis. A minimum of 20,000 cells were analyzed and the positive-stained cells were counted by flow cytometry (FACScalibur). Mouse PE- and FITC- conjugated unspecific antibodies were used as isotope control. The data were analyzed with the Cell Quest Pro Software (BD Bioscience) and expressed as the mean fluorescent intensity (MFI) of positive-stained cells.

### Real-time PCR

B cells plated in 12-well plates (5×10^5^ cells per ml) were treated with DMSO, batyl alcohol or chimyl alcohol (100 nM) in the presence of stimuli for 5 days. Total RNA was isolated from cultured B cells using RNeasy mini kit (Qiagen). 0.5 µg RNA was reverse-transcribed into cDNA using Oligo-dT. Real-time PCR with SYBR Green dye (BioRad) was performed using ABI 7300 system. The Germline (GL) IgG (γ1) primers were: forward, 5′- GGCCCTTCCAGATCTTTGAG-3′; reverse, 5′- GGATCCAGAGTTCCAGGTCACT -3′. The GAPDH mRNA (forward, 5′- TTAGCACCCCTGGCCAAGG-3′; reverse, 5′- CTTACTCCTTGGAGGCCATG-3′) was used as internal control for RNA integrity and RT-PCR amplification. The relative expression level of transcript was determined using standard curve method after GAPDH normalization. The quantitative real-time PCR was performed using the following conditions. Reaction mixtures contained 12.5 µl of SYBR Green I dye master mix (Applied Biosystems), 2 pmol/L each of forward and reverse primers, and 5 µl of 100 times diluted cDNA. PCR reactions were run in triplicate (95°C for 10 min, and 40 cycles of 95°C for 15 s, 58°C for 30 s and 72°C for 30 s).

### ELISA Analysis

To assess cytokine production, T cells plated in 12-well plates (5×10^5^cells per ml) were cultured with DMSO, batyl alcohol or chimyl alcohol (100 nM) in the presence of stimuli for 7 days. Concentrations of TNF-α, IFN-γ, IL-4 and IL-10 in culture supernatants were determined by sandwich ELISA. All of the ELISA assays were performed according to the manufacturer’s instructions. The results were expressed as pg/ml and all assays were run in duplicate with three separate experiments.

### Statistical Analysis

Data were reported as mean ± SE. The main effects of AKGs were evaluated by one-way ANOVA. Differences among groups, *P* value<0.05, were determined by using Bonferroni corrected Post-Hoc Test.

## Results

### AKGs Boost the Proliferation of B Cells

**Figure 1:**
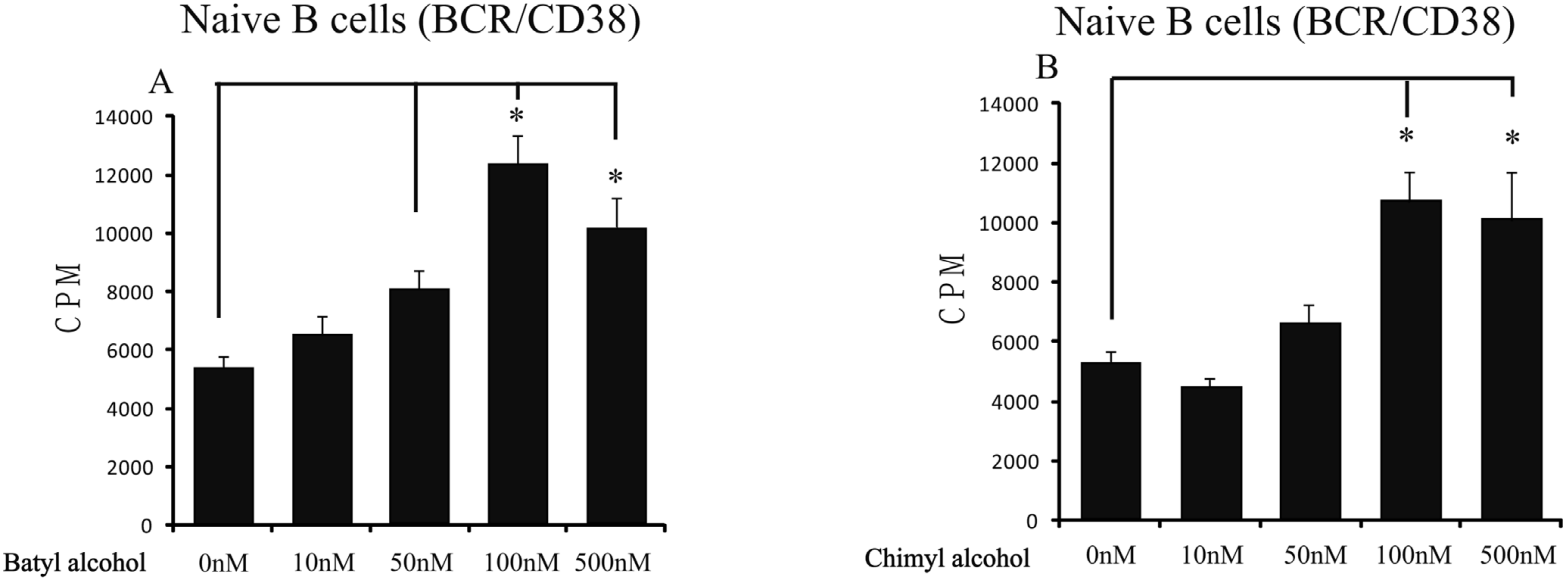
AKGs boosted B cell proliferation stimulated by anti-BCR and anti-CD38. (A–B) B cells were stimulated with anti-BCR and anti-CD38 (each 1 µg/ml), and cultured with different concentrations of batyl alcohol (A) or chimyl alcohol (B) for 72 h. Cells were treated with [^3^H]thymidine (0.2 µCi/well) within the last 4 h of culture, and [^3^H]thymidine incorporation was measured after cell harvest. The data shown represented the mean±SE for three independent experiments. *, P<0.05 versus control (DMSO treatment), Bonferroni corrected Post-Hoc test.

B cells were treated with the combination of 1 µg/ml BCR agonistic antibody (anti-BCR) and CD38 agonistic antibody (anti-CD38) in the absence and presence of batyl alcohol or chimyl alcohol (10 nM to 500 nM). The cell proliferation was determined by [^3^H]-thymidine incorporation assay. The results showed that either batyl alcohol or chimyl alcohol was able to enhance the proliferation of B cells stimulated by anti-BCR and anti-CD38 (Fig. 1A, Fig. 1B). The stimulated B cells showed dose-dependent response to AKGs treatment, with maximal response at the concentration of 100 nM for both batyl alcohol (2.3-fold, p<0.001) and chimyl alcohol (2.0-fold, p = 0.002).

### AKGs Increase the Differentiation of B Cells

**Figure 2:**
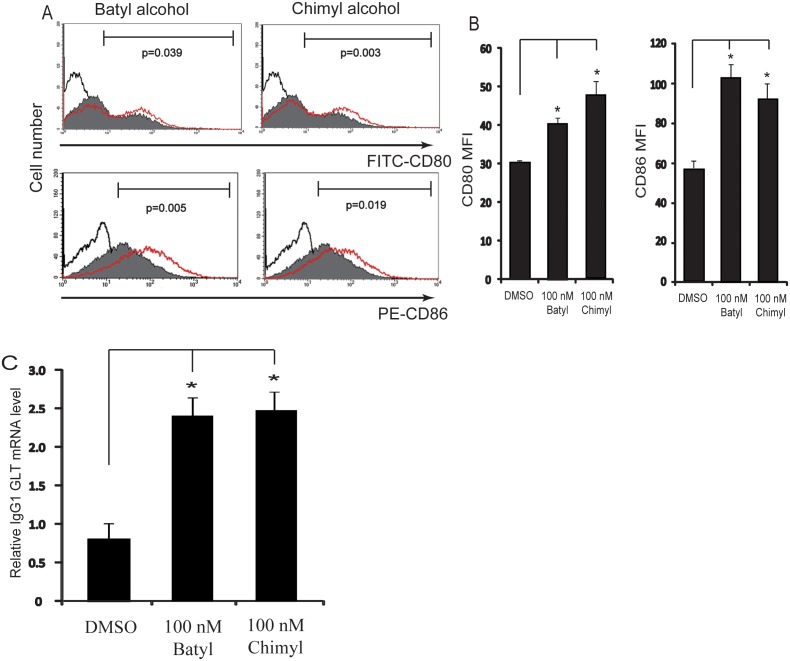
AKGs increased the B cell activation and differentiation stimulated by anti-BCR and anti-CD38. (A) Isolated B cells were stimulated with anti-BCR and anti-CD38 (each 1 µg/ml), and cultured with DMSO, 100 nM batyl alcohol or 100 nM chimyl alcohol for 5 days. Cells were analyzed for the surface markers CD80 and CD86 by flow cytometry, and histogram plots showed the expression of indicated proteins in DMSO (shaded histograms), batyl or chimyl alcohol (red lined histograms) treated B cells. The CD80+ or CD86+ B cells were gated, and the p values for CD80 and CD86 expression differences between control and treated cells were indicated. The black lined histograms indicate isotype control. (B) The graph showed the mean fluorescent intensity (MFI) of CD80 and CD86 within gates in BCR/CD38–stimulated B cells of DMSO, batyl or chimyl alcohol treatment. The data shown represented the mean±SE for at least three independent experiments. (C) Isolated B cells were treated as in panel A. RNA from the cells harvested on day 5 was quantitated for the transcription level of GL IgG (γ1) by qRT-PCR. The data shown represented the mean±SE for three independent experiments. *, P<0.05 versus control (DMSO treatment), Bonferroni corrected Post-Hoc test.

CD80/CD86 costimulators play a key role in the polyclonal B cell activation mediated by CD4+ T cells. In the present study, CD80 and CD86 molecules were used as activation markers for B cells. The splenic B cells were cultured with DMSO, batyl alcohol or chimyl alcohol (100 nM) in the presence of stimuli (anti-CD38 and anti-BCR, each 1 µg/ml) for 5 days. CD80 and CD86 expressions were assessed by flow cytometry after BCR/CD38 stimulation in the presence or absence of AKGs. The results showed that the expressions of CD80 and CD86 on the stimulated B cells were significantly increased by batyl alcohol (p = 0.039 for CD80 and p = 0.005 for CD86) and chimyl alcohol (p = 0.003 for CD80 and p = 0.019 for CD86) (Fig. 2A, 2B). This result suggests that AKGs can increase the potential of naïve B cells to recruit T helper cells and promote T cell-dependent activation.

GL Ig gene transcription is an essential event preceding class-switch recombination, and is considered to be an early feature for B cell maturation. Because AKGs markedly increased B cell proliferative activity, the following experiment was performed to examine whether AKGs also regulate the expression of GL Ig transcripts. The splenic B cells were cultured with DMSO, batyl alcohol or chimyl alcohol (100 nM) in the presence of stimuli (anti-CD38 and anti-BCR, each 1 µg/ml) for 5 days. The expression level of IgG (γ1) GL transcript was assessed to study the effect of AKGs on stimulation-induced B cell maturation. It was found that the level of IgG (γ1) GL transcript was markedly increased by either batyl alcohol (2.9-fold, p = 0.015) or chimyl alcohol (2.9-fold, p = 0.013) as shown in Fig. 2C.

### AKGs Boost the Proliferation of T Cells

**Figure 3:**
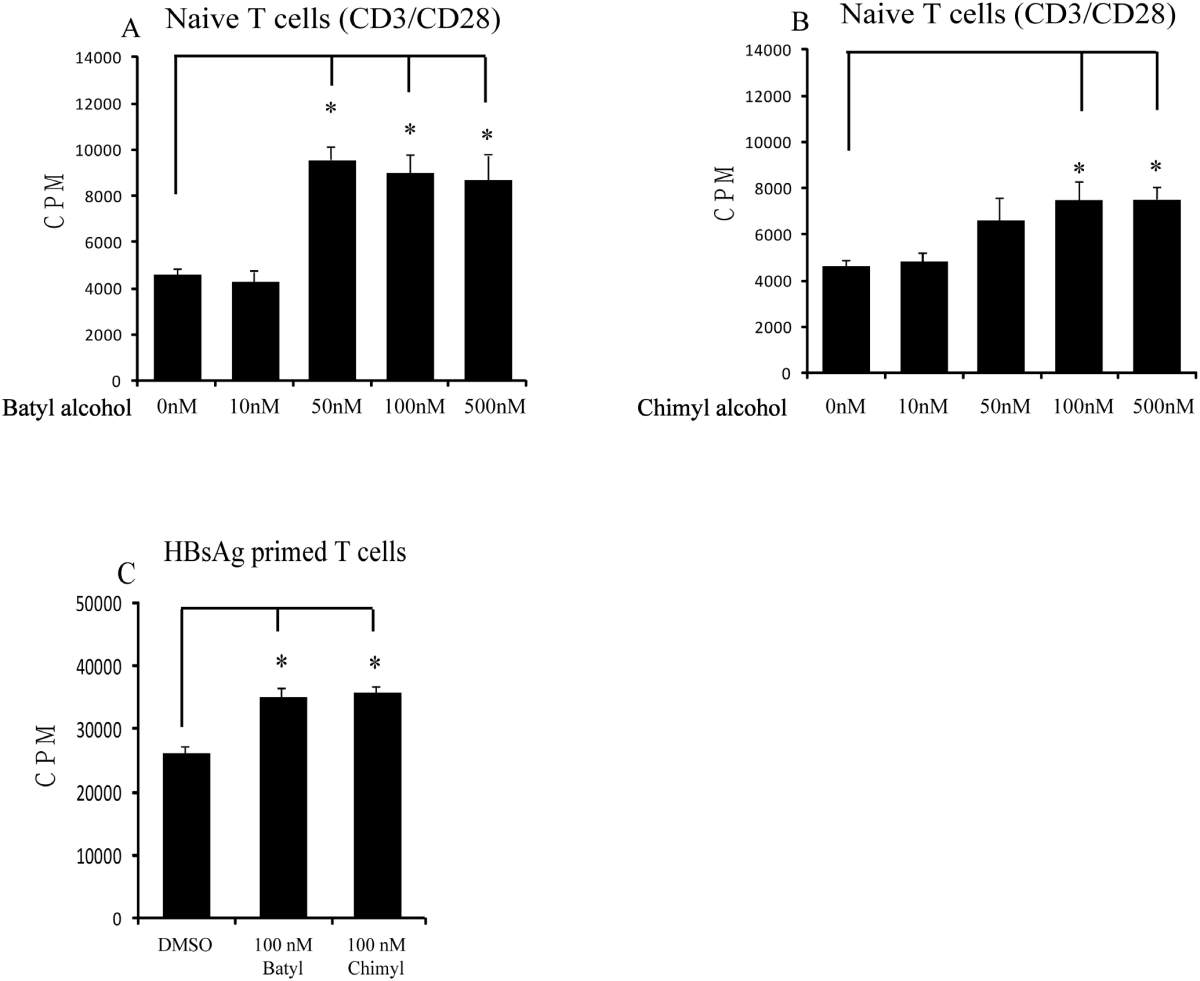
AKGs boosted the proliferation of T cells induced by anti-CD3 and anti-CD28 or by antigen –bearing DCs. (A–B) Naïve CD4+CD62L+ T cells were stimulated with anti-CD3 and anti-CD28 (each 1 µg/ml), and cultured with different concentrations of batyl alcohol (A) or chimyl alcohol (B) for 72 h. Cells were treated with [^3^H]thymidine (0.2 µCi/well) within the last 4 h of culture, and [^3^H]thymidine incorporation was measured after cell harvest. (C) Whole CD4+ T cells (from HBsAg immunized mice) were stimulated with HBsAg –bearing DCs, and cultured with batyl alcohol or chimyl alcohol (100 nM) for 72 h. [^3^H]thymidine was determined as in A and B. The data shown represented the mean±SE for three independent experiments. *, P<0.05 versus control (DMSO treatment), Bonferroni corrected Post-Hoc test.

Naïve CD4+ cells (CD4+CD62L+) were treated with anti-CD3 and anti-CD28 (each 1 µg/ml) in the absence and presence of batyl alcohol or chimyl alcohol (10 nM to 500 nM), and cell proliferative activity was measured. The [^3^H]thymidine incorporation assay showed that AKGs significantly increased CD3/CD28-induced T cell proliferation, with maximal effects at the concentration of 50 nM (2.1-fold, p<0.001) and 100 nM (1.6-fold, p = 0.019) for batyl alcohol and chimyl alcohol respectively (Fig. 3A, 3B). To study whether AKGs have the capability to enhance the memory T cells response, the total CD4+ cells were co-incubated with HBsAg -bearing DCs in the absence and presence of batyl alcohol or chimyl alcohol (100 nM). Similar enhancement on HBsAg –stimulated T cell proliferation was observed when cells were treated with either batyl alcohol (1.4-fold, p = 0.022) or chimyl alcohol (1.5-fold, p = 0.01) (Fig. 3C). Therefore, AKGs were able to enhance not only the naïve T cell response but also the memory T cell response.

### AKGs Increase the Differentiation of T Cells

**Figure 4:**
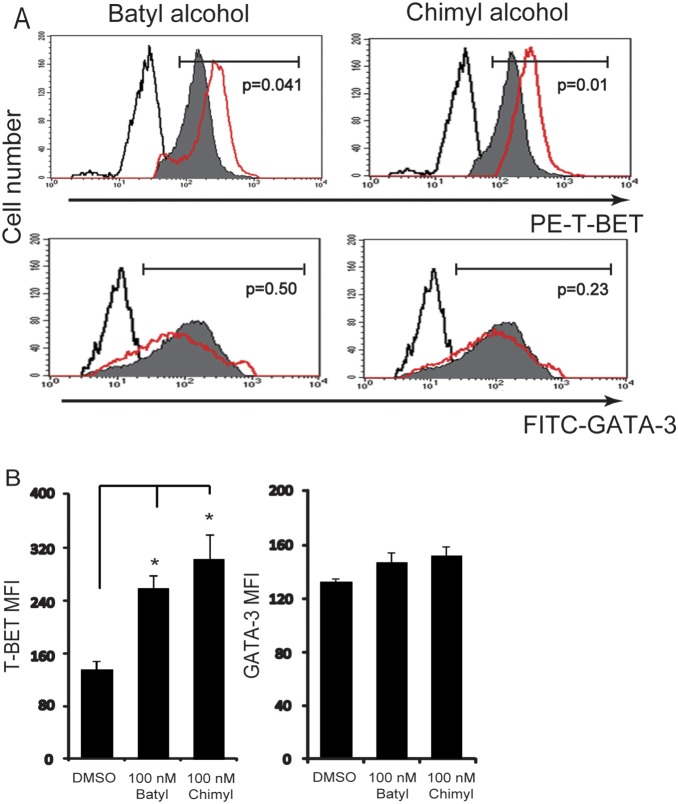
AKGs shaped the differentiation of naïve T cells induced by anti-CD3 and anti-CD28. (A) CD4+CD62L+ T cells were stimulated with anti-CD3 and anti-CD28 (each 1 µg/ml), and cultured with DMSO, 100 nM batyl alcohol or 100 nM chimyl alcohol for 7 days. Th1 transcription factor T-BET and Th2 transcription factor GATA-3 were analyzed by flow cytometry, and histogram plots showed the expression of indicated proteins in DMSO (shaded histograms), batyl or chimyl alcohol (red lined histograms) treated cells. The T-BET+ or GATA-3+ T cells were gated, and the p values for T-BET and GATA-3 expression differences between control and treated cells were indicated. The black lined histograms indicate isotype control. (B) The graph showed the mean fluorescent intensity (MFI) of T-BET and GATA3 within gates in CD3/CD28–stimulated T cells of DMSO, batyl or chimyl alcohol treatment. The data shown represented the mean±SE for at least three independent experiments. *, P<0.05 versus control (DMSO treatment), Bonferroni corrected Post-Hoc test.

**Figure 5:**
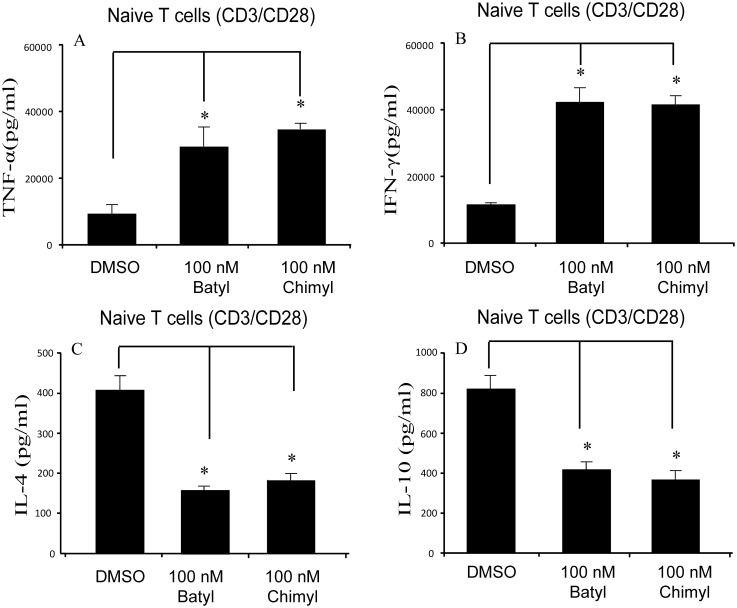
AKGs modulate Th1/Th2 cytokine production of naïve T cells induced by anti-CD3 and anti-CD28. (A–D) CD4+CD62L+ T cells were stimulated with anti-CD3 and anti-CD28 (each 1 µg/ml), and cultured with DMSO, 100 nM batyl alcohol or 100 nM chimyl alcohol for 7 days. The supernatants were collected and analyzed for cytokines by ELISA. (A): TNF-α; (B): IFN-γ; (C): IL-4; (D): IL-10. The data shown represented the mean±SE for three independent experiments. *, P<0.05 versus control (DMSO treatment), Bonferroni corrected Post-Hoc test.

Naïve CD4+/CD62L+ T cells were stimulated using anti-CD3 and anti-CD28. 100 nM batyl alcohol or chimyl alcohol was applied to the medium in the treatment groups, and DMSO was used as the mock in the non-treatment groups. Th1 transcription factor T-BET and Th2 transcription factor GATA-3 were assessed by flow cytometry after 7d incubation. The results showed that the T-BET expression but not GATA-3 expression was increased by AKGs treatment in CD3/CD28–stimulated T cells (Fig. 4). Similar effects of AKGs on the expression of T-BET/GATA-3 were observed in HBsAg –stimulated T cells (Fig. S1). Meanwhile, Th1 characteristic cytokines (TNF-α and IFN-γ) and Th2 characteristic cytokines (IL-4 and IL-10) were assessed by ELISA after 7d incubation (Fig. 5). The results showed that Th1 characteristic TNF-α and IFN-γproductions were increased by AKGs treatment in CD3/CD28–stimulated T cells. However, Th2 characteristic IL-4 and IL-10 productions were decreased by AKGs treatment in CD3/CD28–stimulated T cells. In HBsAg –stimulated T cells, similar effects on Th1/Th2 cytokine productions were observed after AKGs treatment (Fig. S2).

## Discussion

AKGs have been demonstrated to be an effective adjuvant for antigen response. Acevedo et al reported that synthetic AKGs enhanced anti-ovalbumin response in mice [10]. However, they have not clarified whether antigen response stimulated by AKGs was T-cell dependent or T-cell independent. Most antigens are T-dependent, meaning T cell help is important for antibody production. Neonates often have weak response to vaccination as compared to older children and adults, which is mainly attributed to poor coordination of immune cells [14]. Maximal antibody production and memory generation require both B cell and T cell activation [15]. Since AKGs were considered as a crucial immune factor in breast-milk and a promising adjuvant for vaccination response [4], [10], it is important to characterize how AKGs can regulate the antigen stimulated response of immune cells, especially the process of proliferation and activation. Therefore, we studied the immunomodulatory effects of AKGs on the proliferation and activation of splenic lymphocytes using *in-vitro* cultured model sensitized by antigen. In the present study, 100 nM AKGs was found to be the optimal concentration to enhance the stimulation induced cell response, and this amount approximately equals to 1∶1000 dilution of human breast-milk [4]. Several previous studies applied 1∶100∼1∶1000 dilution of breast-milk to treat cell lines [16], [17], and the AKGs dosage in our study was comparable to that normally accepted by peers. AKGs markedly increased the stimulatory effect of anti-BCR and anti-CD38 on the proliferation of B cells. BCR stimulation, which mimics the antigen binding event, leads to a remarkable increase in B cell proliferation. CD38, which functions as an accessory receptor for the BCR, can increase the pyridine nucleotide (NAD) metabolism required for B cell activation [18]. This result demonstrated that AKGs treatment is able to promote B cell clonal proliferation which is a critical step for sufficient immune response.

It is well known that B cells undergo isotype switching recombination and activation soon after stimulation. GL Ig expression initiates the process of class switching recombination and is considered to be an essential event for B cell maturation [19]. We demonstrated in our study that AKGs markedly increased the stimulation-induced IgG (γ1) GL expression. Our experiments also demonstrated that AKGs could increase B cell surface expressions of CD80 (B7-1) and CD86 (B7-2). CD80 and CD86 are usually expressed on antigen-presenting cells (DCs, macrophages and B cells), and are required for T cell-dependent B cell activation [20]. Previous study provided solid evidence that B7/CD28–mediated signaling regulates B cell responses [21]. Mice deficient in CD80 and CD86 lack the formation of germinal center and induce only limited Ig class-switch recombination, memory formation and affinity maturation [22]. B7 costimulation was also shown to influence IgG production *in vivo* [23]. All of these lines of evidence support the hypothesis that AKGs favor the maturation and activation of B cells after stimulation.

CD4+ T cells are of crucial importance for humoral and cellular immunity, and they may differentiate into Th1, Th2, Th17 or Treg phenotypes, with distinct immunological functions and characteristic cytokine products [24]. CD4+ T cells are activated by antigen presented by MHCII molecule on antigen-presenting cells, and then secrete cytokines to activate B cells [25]. We examined the effects of AKGs on the proliferation of T cells driven by anti-CD3/CD28 antibody or antigen -pulsed DCs. The results showed that naïve CD4+CD62L+ T cells responded to the CD3/CD28 stimulation with a higher rate of proliferation in the presence of AKGs. Meanwhile, upon the specific antigen stimulation, AKGs also showed similar effect on the proliferation of CD4+ T cells. CD3/CD28 activation promotes a number of signaling cascades that ultimately leads to cytokine production, proliferation and differentiation. The activated TCR/CD3 complex recruits and phosphorylates downstream inducible T cell kinase (ITK) and Vav [26], [27]. ITK can phosphorylate phospholipase C γ1 (PLCγ1) and produce the second messengers inositol trisphosphate (IP3) and diacylglycerol (DAG) [28]. DAG is able to activate PKC and the MAPK/ERK pathways, which can both promote NF-κB signaling activation. IP3 is able to trigger the influx of calcium which results in T cell activation [29]. It was known that AKGs are involved in the synthesis of 1-O-alkylcerophosphocholine, the precursor of platelet-activating factor (PAF), and increase the production of ether analogue of DAG in monocyte cell line. However, the ether analogue of DAG originated from AKGs has been demonstrated to have inhibiting activity on PKC in endothelial cells [30], [31]. Thus, the mechanism of action of AKGs remained to be particularly unclear. Interestingly, Pedrono *et al* showed that AKGs can modulate the permeability of voltage-gated calcium channels, thus increase the cytosolic calcium influx in Jurkat T cells. Calcium influx through voltage-gated calcium channels is crucial for intracellular Ca2+ rise after antigen receptor activation, and triggers signal for T cell proliferation and differentiation during immune response [32].

Type 1 (Th1) and type 2 (Th2) T-helper cells have been demonstrated to play an essential role in regulating adaptive immune response during allergy, inflammation and infection. The pathogenic role of Th1/Th2 balance has been described for certain autoimmune diseases such as insulin-dependent diabetes mellitus and rheumatoid arthritis [33], [34]. The beneficial effects of SLO on Th1/Th2 unbalanced diseases have been well documented [35]. It is also noted that breastfeeding has protective effect against allergic and autoimmune diseases [36]. In the present study, AKGs, the bioactive constituents in both SLO and human milk, were tested for their impact on Th1/Th2 balance in T helper cell activation process. Th1/Th2 polarized cell subsets were identified by T-BET/GATA-3 transcription factors and their signature cytokines. According to the previous study, changes in the ratio of T-BET/GATA-3 transcription factors reflected changes in the Th1 cytokines (TNF-α and IFN-γ) and Th2 cytokines (IL-4 and IL-10) [37]. Previous research showed that Th1 indicators were elicited by AKGs administration as shown by the increase in IgG2a/IgG1 ratio and IL-12 level [10]. Our results confirmed the previous reports that AKGs induced the production of Th1 cytokines (TNF-α and IFN-γ) as well as Th1–related gene (T-BET) expression upon CD3/CD28 and HBsAg stimulation. However, AKGs were found in our study to inhibit the Th2 response as shown by the decrease of cytokines (IL-4 and IL-10), which was not consistent with previous result using IgG1 as Th2 indicator [10]. Overall, this study showed that AKGs could provide an environment for Th1 polarization under not only non-specific agonist stimulation but also specific antigen stimulation.

## Conclusions

In summary, our experiments provide evidence that AKGs enhance the proliferation and activation of mouse B lymphocytes upon non-specific BCR/CD38 stimulation, and promote the proliferation and Th1 differentiation of naïve and activated/memory T-cell subsets. These results obtained from cultured lymphocytes give a possible explanation about how AKGs could promote immunity response *in vivo.*

## Supporting Information

Figure S1 **AKGs shaped the differentiation of memory T cells induced by antigen (HBsAg) –bearing DCs.** (A) Whole CD4+ T cells (from HBsAg immunized mice) were stimulated with HBsAg –bearing DCs, and cultured with DMSO, 100 nM batyl alcohol or 100 nM chimyl alcohol for 7 days. Th1 transcription factor T-BET and Th2 transcription factor GATA-3 were analyzed by flow cytometry, and histogram plots showed the expression of indicated proteins in DMSO (shaded histograms), batyl or chimyl alcohol (red lined histograms) treated cells. The T-BET+ or GATA-3+ T cells were gated, and the p values for T-BET and GATA-3 expression differences between control and treated cells were indicated. The black lined histograms indicate isotype control. (B) The graph showed the mean fluorescent intensity (MFI) of T-BET and GATA3 within gates in antigen (HBsAg) –stimulated T cells of DMSO, batyl or chimyl alcohol treatment. The data shown represented the mean±SE for at least three independent experiments. *, P<0.05 versus control (DMSO treatment), Bonferroni corrected Post-Hoc test.
(TIF)

Figure S2 **AKGs modulate Th1/Th2 cytokines production of memory T cells induced by antigen (HBsAg) –bearing DCs.** (A-D) Whole CD4+ T cells (from HBsAg immunized mice) were stimulated with HBsAg –bearing DCs, and cultured with DMSO, 100 nM batyl alcohol or 100 nM chimyl alcohol for 7 days. The supernatants were collected and analyzed for cytokines by ELISA. (A): TNF-α; (B): IFN-γ; (C): IL-4; (D): IL-10. The data shown represented the mean±SE for three independent experiments. *, P<0.05 versus control (DMSO treatment), Bonferroni corrected Post-Hoc test.
(TIF)
