# Supplementary figures and images for: Alkylglycerols Modulate the Proliferation and Differentiation of Non-Specific Agonist and Specific Antigen-Stimulated Splenic Lymphocytes
Source: PLoS One. 2014 Apr 24;9(4):e96207. doi: 10.1371/journal.pone.0096207 (PMC3999215; doi:10.1371/journal.pone.0096207)

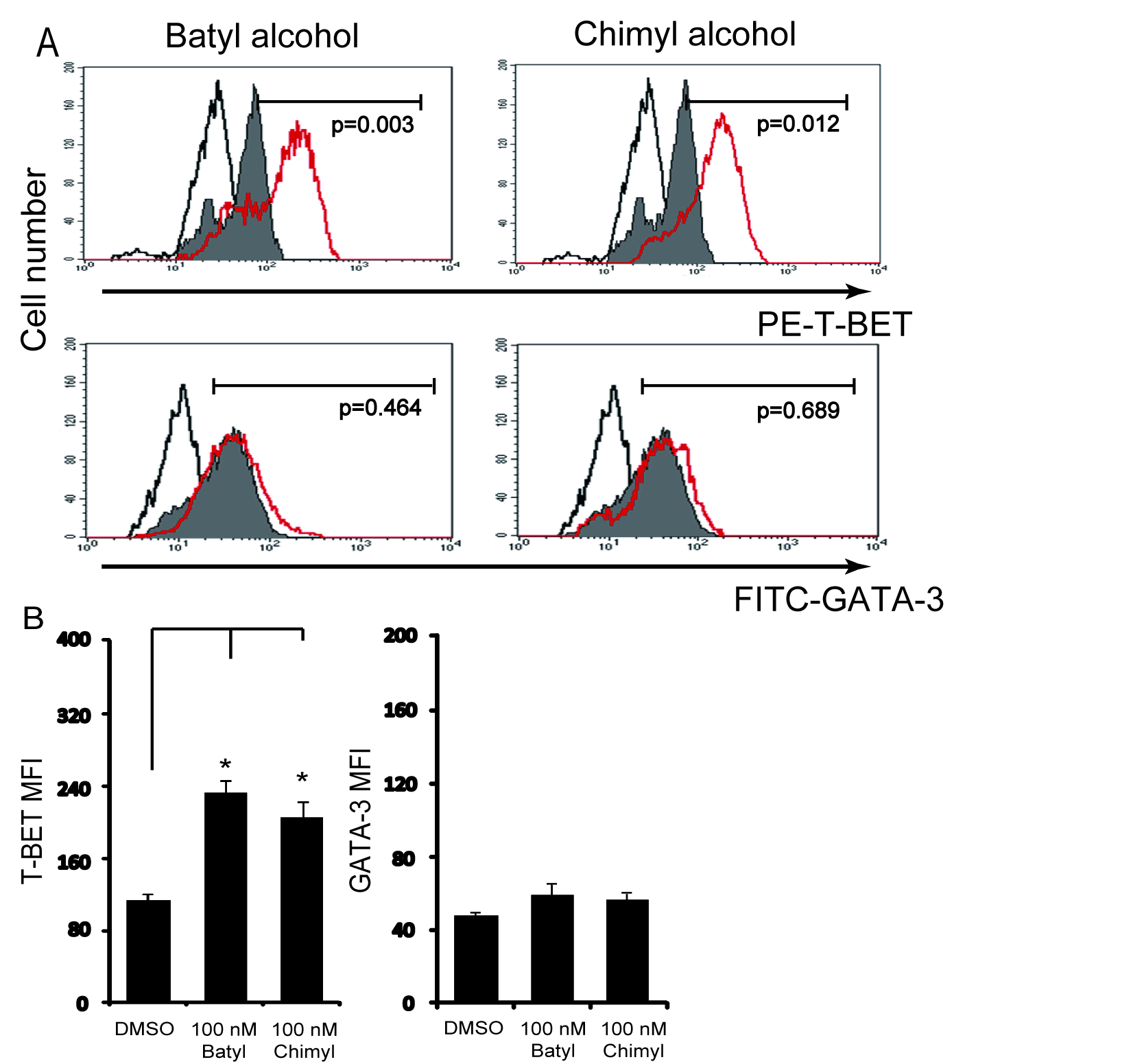

Supplement: Figure S1 — AKGs shaped the differentiation of memory T cells induced by antigen (HBsAg) –bearing DCs. (A) Whole CD4+ T cells (from HBsAg immunized mice) were stimulated with HBsAg –bearing DCs, and cultured with DMSO, 100 nM batyl alcohol or 100 nM chimyl alcohol for 7 days. Th1 transcription factor T-BET and Th2 transcription factor GATA-3 were analyzed by flow cytometry, and histogram plots showed the expression of indicated proteins in DMSO (shaded histograms), batyl or chimyl alcohol (red lined histograms) treated cells. The T-BET+ or GATA-3+ T cells were gated, and the p values for T-BET and GATA-3 expression differences between control and treated cells were indicated. The black lined histograms indicate isotype control. (B) The graph showed the mean fluorescent intensity (MFI) of T-BET and GATA3 within gates in antigen (HBsAg) –stimulated T cells of DMSO, batyl or chimyl alcohol treatment. The data shown represented the mean±SE for at least three independent experiments. *, P<0.05 versus control (DMSO treatment), Bonferroni corrected Post-Hoc test. (TIF) [file pone.0096207.s001.tif]

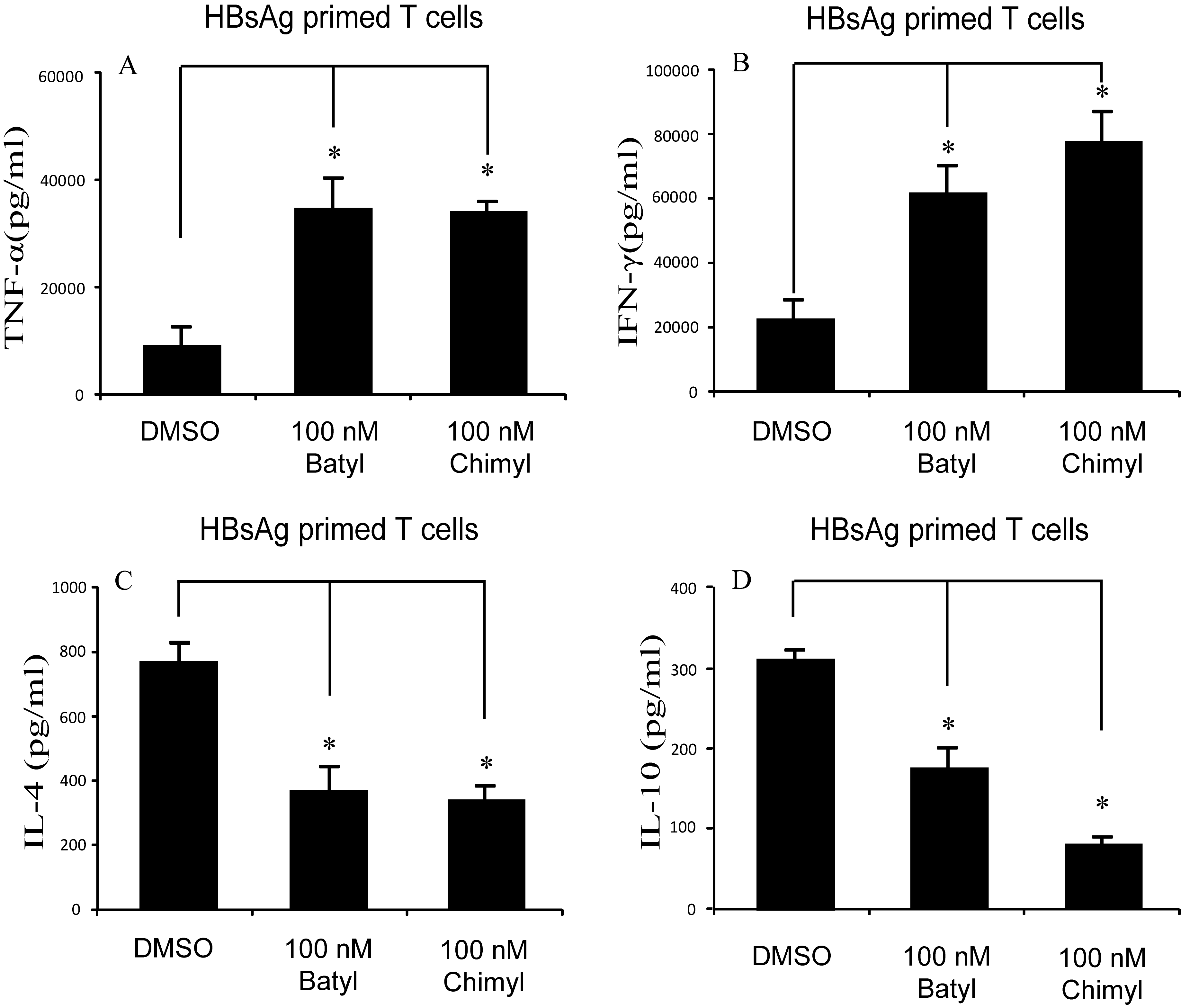

Supplement: Figure S2 — AKGs modulate Th1/Th2 cytokines production of memory T cells induced by antigen (HBsAg) –bearing DCs. (A-D) Whole CD4+ T cells (from HBsAg immunized mice) were stimulated with HBsAg –bearing DCs, and cultured with DMSO, 100 nM batyl alcohol or 100 nM chimyl alcohol for 7 days. The supernatants were collected and analyzed for cytokines by ELISA. (A): TNF-α; (B): IFN-γ; (C): IL-4; (D): IL-10. The data shown represented the mean±SE for three independent experiments. *, P<0.05 versus control (DMSO treatment), Bonferroni corrected Post-Hoc test. (TIF) [file pone.0096207.s002.tif]
